# Student-Reported Cognitive Engagement with Structured Generative-AI Questioning: Differentiated Associations with AI, Teacher, and Peer Questions

**DOI:** 10.3390/bs16101814

**Published:** 2026-10-04

**Authors:** Yuqiang Liu, Zehua Ji, Siguang Zhang

**Affiliations:** 1 School of Economics and Management, North University of China, Taiyuan 030051, China; sz202509022@st.nuc.edu.cn; 2 Institutes of Science and Development, Chinese Academy of Sciences, Beijing 100190, China

**Keywords:** generative AI, cognitive engagement, critical thinking, classroom questioning, higher education

## Abstract

Generative AI can sustain follow-up questioning, but questions do not constitute engagement by themselves. This qualitative-dominant mixed-methods study examined student-reported cognitive engagement around AI, teacher, and peer questions in a Chinese university critical-thinking course. Seven reflection points produced 1034 matched submissions, including 648 substantive texts. A structured source item provided an implementation check for 399 responses at two common reflection points, and two human coders independently analyzed a stratified sample of 120 reflections. Sampling-weighted and reflection-stratified checks assessed sensitivity to unequal stratum sizes. Within this structured intervention, AI-salient accounts more often concerned assumptions, criteria, evidence, uncertainty, and reason organization; teacher-salient accounts made situated clarification more visible; and the smaller peer-salient set highlighted comparison and counter-positioning. These patterns changed little after weighting, although the AI prompt explicitly requested several of the functions later observed in AI-salient reflections. Across sources, reason organization was clearer than low-pressure participation. The reflections did not establish inherent source effects, complete interaction sequences, critical-thinking gains, or a consistently stronger hybrid pattern. They support a testable proposition: question sources may contribute complementary reasoning resources when students remain responsible for evaluating and revising their judgments.

## 1. Introduction

Critical-thinking courses ask students to explain reasons, test assumptions, consider alternatives, and revise a position when evidence warrants it (King, 1995; Paul & Elder, 2019). Questions can make gaps in reasoning visible, but a prompt alone does not determine what students do with it. Learning depends on whether they elaborate an answer, compare it with another position, monitor its limits, or use it to revise a judgment (Alexander, 2020; Nystrand et al., 2003).

This distinction matters because student engagement is multidimensional. Behavioral engagement concerns observable participation and persistence, emotional engagement concerns affective involvement, and cognitive engagement concerns students’ investment in understanding and strategic thought (Fredricks et al., 2004; Kahu, 2013). Students may also contribute agentically by shaping the flow of instruction (Reeve & Tseng, 2011). These dimensions are related but cannot be inferred from one another. A student can speak often without examining a claim or reason carefully without reporting greater confidence or public participation. The present study therefore uses a deliberately narrow definition: cognitive engagement refers to reflected effort to elaborate, compare, qualify, organize, and monitor reasons. It does not stand for attendance, participation volume, enjoyment, psychological safety, or durable learning.

Generative AI adds a rapid and repeatable source of follow-up questions. It can request reasons, expose assumptions, generate counter-positions, and ask about evidence or consequences (McGrath et al., 2025). Yet technical capability does not establish an educational role (Bearman et al., 2023). What students encounter also depends on prompt design: a system instructed to probe evidence and assumptions is likely to make those functions more visible. Guo et al. (2025) found that the relationship between GenAI use and student engagement varied across learning contexts and with the balance between challenge and support. Other classroom studies show that teacher mediation is needed to turn AI output into an object for evaluation rather than a ready-made answer (Borges et al., 2026), while a comparison of generative-AI feedback and peer feedback suggests that each source supplies different resources for evaluative judgment (Xie et al., 2025). A plausible AI question is therefore only an invitation to engage. Students must decide what deserves attention and whether the resulting answer should be accepted, revised, or rejected.

This problem is often framed as a choice between human and AI support. That framing obscures the distribution of reasoning work. Teachers can interpret an incomplete answer in context, select what deserves public attention, and connect a contribution to the purpose of the lesson. Peers can require comparison, explanation, and response to another person’s standpoint when the task demands more than parallel group work (He et al., 2025; Webb et al., 2019). AI can provide immediate and persistent prompts, but it does not share the classroom history or determine which issue has educational or social importance in the moment. The design problem is thus not simply whether AI increases engagement. It is which forms of cognitive work become visible around each question source and how those forms can be connected without delegating judgment to the system.

This question is especially relevant in higher education, where students can use general-purpose GenAI with little institutional mediation. The Chinese case examined here is not treated as representative of a uniform national or regional classroom. It offers a bounded setting in which teacher authority, small-group discussion, and critical-thinking goals already had established places. University enrollment alone does not ensure growth in critical thinking (Loyalka et al., 2021), and course tasks must specify how criticality is practiced (Dispa et al., 2025). A course that deliberately varies the main source of follow-up questions can therefore show not whether one source is universally better, but what students notice each source asking them to do.

Reflection cards provide situated evidence for this purpose. They record what students selected and made meaningful after a classroom activity; they do not reproduce the whole activity. Their content is shaped by the prompt, timing, and classroom context (Christensen & Weissmann, 2025; Sheridan et al., 2025), and reflective statements should not be treated automatically as evidence of metacognitive development or improved critical thinking (Pousi et al., 2025). Used conservatively, however, they can reveal the question source students remembered, the reasoning work they described, and the limits they attached to that experience.

This study analyzed reflection cards from a first-year course entitled Critical Thinking. The course included teacher-led Socratic questioning, AI-supported questioning, and a hybrid arrangement in which small groups used AI-generated follow-up questions before teacher extension. The study did not test treatment effects or improvement in critical-thinking outcomes. It examined how the deliberately structured arrangements shaped remembered source salience, which forms of cognitive engagement students associated with AI, teacher, and peer questions, and where their accounts placed the limits of these roles. Its contribution is an intervention-specific source-function account of student-reported cognitive engagement, not a ranking of classroom conditions or questioners.

The study addressed three research questions:

1. What remembered question-source profiles appeared across the three classroom arrangements at the two common reflection points?
2. Within these structured questioning arrangements, what forms of cognitive engagement and related experience did students associate with AI-, teacher-, peer-, and mixed-source questions?
3. How did maximum-variation cases explain the observed source-function associations, possible complementarity, and limits of these question sources?

## 2. Conceptual Framework: Questioning Roles and Cognitive Engagement

### 2.1. Source, Function, and Reported Cognitive Work

Socratic questioning can request clarification, reasons, evidence, assumptions, alternatives, consequences, or acknowledgment of uncertainty (Paul & Elder, 2019; Yang et al., 2005). These functions describe what a question asks for; they do not show whether a learner engaged with it. The ICAP framework distinguishes passive receipt from active, constructive, and interactive engagement, with constructive activity involving the generation of inferences or explanations and interactive activity involving contributions that build on one another (Chi & Wylie, 2014). Because reflection cards are retrospective rather than observational, they were not used to classify students into ICAP levels. The framework instead helps distinguish reflected cognitive work, such as elaborating criteria, testing assumptions, comparing positions, and reorganizing reasons, from the mere presence of an AI tool or a question.

Dialogic teaching further places questions within sequences of response and uptake (Alexander, 2020; Nystrand et al., 2003). The analysis accordingly separates three objects: source, or who was remembered as asking; function, or the reasoning operation described; and reported cognitive work, or what the student said the question led them to examine or reorganize. Reflection cards provide direct evidence about remembered source and described function. They provide partial, retrospective evidence about cognitive work and only limited evidence about uptake because they are not transcripts of complete exchanges.

### 2.2. Source-Linked Functions and Retained Judgment

AI, teachers, and peers may ask overlapping questions without taking the same pedagogical role. AI can help expand an initial position, but learners must monitor, verify, and sometimes reject its contribution (Hwang & Lee, 2025; Kim et al., 2025). Hybrid-intelligence research similarly treats educational value as a product of structured relations between automated support and human inquiry (Chen et al., 2026). Excessive delegation may lower mental effort while weakening depth or monitoring (Fan et al., 2025; Stadler et al., 2024). Teachers can add contextual judgment and pedagogical authority, while peers can add a social setting for comparison and explanation. These are design possibilities rather than fixed properties of each source.

The term questioning ecology names this bounded distribution of reasoning resources; it is an analytic description rather than a validated classroom model. Orchestration refers to the design possibility that contributions from different sources inform later reasoning. Reflection cards can show perceived complementarity, but a complete orchestration sequence would require interaction records.

## 3. Materials and Methods

### 3.1. Research Design and Course Context

This study used a qualitative-dominant convergent mixed-methods design. A descriptive quantitative strand established reflection-card coverage and remembered question-source salience. A qualitative strand used multi-label coding and maximum-variation cases to examine the cognitive work, experiences, and limits students associated with each source. The two strands addressed different parts of the same research questions and were integrated by weaving aggregate source patterns with coded functions and case-level evidence (Johnson et al., 2007; Fetters et al., 2013). The design did not estimate comparative effects on critical-thinking outcomes.

The study was embedded in an eight-week, 16_h undergraduate course entitled Critical Thinking at a public university in Shanxi Province, central China. Participants were first-year students from two economics and management program clusters across six intact classes. Programme 1 contributed two AI-supported classes (39 and 38 matched reflection contributors) and one teacher-led class (36 contributors); Programme 2 contributed one teacher-led class (37 contributors) and two hybrid classes (35 and 36 contributors). Nine upper-year students repeating or supplementing the course were excluded. The matched reflection corpus represented 221 first-year students. No demographic variables beyond those required for roster matching and programme classification were used in this analysis. The classes were assigned to existing classroom arrangements; condition membership was not treated as individual random assignment, and arrangement was partly confounded with programme. The first author taught all six classes using the common course materials. This held the instructor constant across arrangements, although it did not remove programme or intact-class confounding. Class and instructor allocation are reported in Supplementary Table S5.

### 3.2. Questioning Conditions and Classroom Implementation

Weeks 2–7 used issue-based tasks requiring students to state a position, give reasons, consider counter-positions, and identify conditions under which their view might change. Topics included AI writing, autonomous-vehicle ethics, campus speech boundaries, online anonymity, public-health measures, and intergenerational fairness. In the teacher-led condition (T), the instructor supplied the main follow-up questions using Socratic questioning principles. These questions responded to student answers and the immediate classroom discussion. The instructor worked from the same weekly task and broad reasoning goals but did not use a verbatim version of the AI prompt. In the AI-supported condition (A), groups used a standard prompt instructing generative AI to ask questions rather than provide answers. In the hybrid condition (H), groups completed one or two AI-supported cycles and selected a difficult question for instructor follow-up. The instructional purpose was to vary the main source of follow-up questions while retaining the same broad reasoning tasks.

Students in A and H mainly used DeepSeek, Doubao, and Tongyi Qianwen, widely available Chinese-language conversational systems. Platform choice was not assigned or retained at the response level, and the study did not compare systems. A common prompt requested clarification, reasons, assumptions, evidence, counter-positions, consequences, and uncertainty, while prohibiting direct answers. The full bilingual prompt is provided in Supplementary Methods S3, and the weekly tasks are listed in Supplementary Table S3. Implementation evidence consisted of the archived teaching plans, the common AI prompt, and the structured remembered-source item. Complete AI logs, teacher-discourse records, and independent fidelity ratings were not retained. The analysis therefore concerns remembered questioning within this intervention rather than the quality, frequency, or protocol adherence of every question. Peer discussion occurred in all arrangements; remembered AI, teacher, and peer sources are not synonyms for A, T, and H membership.

### 3.3. Reflection Cards and the Analytic Corpus

Short reflection cards were administered at seven points. The first followed course orientation. Later cards contained a structured item asking which source had posed the most thought-provoking question and open fields asking students to record that question, explain why it mattered, describe any change or qualification in their position, consider another standpoint, and comment on the activity. These prompts explicitly elicited reasons, qualification, and alternative standpoints; the resulting codes describe what students supplied in response to those prompts rather than wholly spontaneous behavior. Wording varied slightly with the issue; Supplementary Table S4 maps the recurring prompts to their analytic use. Reflection 2 was an additional teacher-condition exercise on short-video credibility and was not the common Week 2 task listed in the archived teaching-plan sequence. Collection depended on end-of-class time and did not form a balanced weekly panel. The unit of analysis was the submitted reflection, and the same student could contribute at more than one point. Repeated submissions were not modeled as independent observations in inferential tests because no such tests were conducted. No missing cards were imputed. The cards were treated as situated accounts rather than complete interaction records (Christensen & Weissmann, 2025; Sheridan et al., 2025).

The raw files contained 1072 submissions, of which 670 met the substantive-text rule before eligibility matching. Matching to the first-year roster retained 1034 submissions from 221 students. A submission was substantive when an open-text field contained an interpretable clause beyond empty, one-word, “none”, or “no change” entries; 648 matched submissions met this rule. Reflection 4 (campus speech boundaries) yielded 205 matched and 147 substantive texts. Reflection 6 (public-health measures and individual choice) yielded 194 matched and 144 substantive texts. These were the only points with strong coverage in all three conditions and formed the common comparison corpus (Table 1).

A structured item recorded the most salient question source and supplied source-salience results for all matched Reflection 4 and 6 responses (Table 2). Open-text fields supplied evidence about function, experience, and role boundaries. Coders used the structured item for remembered source and required textual evidence for each function or experience code. The form placed the recalled-question and why-it-mattered fields directly after the source item, creating a questionnaire-level link between source and reported cognitive work. When a student did not name the source again in open text, the link rests on that form structure rather than an independent linguistic attribution. Source salience was therefore a perception measure, not an observation of exposure, question frequency, or question quality.

### 3.4. Stratified Coded Sample

From the 291 substantive Reflection 4 and 6 texts, a stratified sample of 120 (41.2%) was drawn: 20 from each reflection-by-condition cell. The sample represented 97 unique students; 23 contributed one selected record at both Reflection 4 and Reflection 6, while 74 contributed at one point only. Accordingly, 46 records came from the 23 students represented at both points, and the remaining 74 records came from 74 students represented at one point. Random sampling within cells used the fixed seed 20260708. The six frame sizes were 53, 52, 42, 53, 53, and 38. This design balanced classroom arrangements, not remembered source categories. Each coded record was therefore assigned the inverse sampling weight *N_h/n_h*, where *N_h* was its stratum’s substantive-text count and *n_h* = 20. Unweighted counts were retained for audit, while weighted percentages estimated patterns in the 291-record substantive corpus. Reflection-stratified estimates were also calculated for the AI- and teacher-salient groups. After codebook refinement and human reconciliation, the same 120 records formed the final coded corpus.

Names, student numbers, class and subgroup labels, and exact submission times were removed. Internal identifiers supported linkage and audit; published quotations use neutral labels Q1–Q6.

### 3.5. Codebook Development and Human Coding

The final multi-label codebook distinguished question source, questioning function, student experience, and role boundary. Function codes included clarification, reasons, evidence, assumptions, counter-positioning, consequences, value conflict, uncertainty, and expression. Experience codes covered expression safety, low-pressure trial, perspective taking, reason organization, continued questioning, perceived limitations, dependence, pressure, and peer support. Boundary codes identified source roles, risks, and perceived complementarity (Supplementary Table S1). Coding was conducted in Chinese.

Codebook v1 was developed from the research questions, reflection fields, and initial reading of the corpus. An AI-assisted preparatory pass was conducted in OpenAI Codex using the GPT-5 model to flag possible ambiguities and organize a disagreement template. Only manually selected Chinese excerpts were entered after names, student numbers, contact details, class and subgroup labels, exact timestamps, and re-linkage keys had been removed; no raw data file or source dataset was uploaded. Processing was subject to the standard privacy and data-retention terms applicable to the authors’ OpenAI account at the time, and no claim of provider-side zero retention is made. Risk control therefore rested on de-identification before transmission and exclusion of direct identifiers and re-linkage keys. The only research copy retained by the authors was the resulting preparatory file in the restricted local project archive; it was excluded from the analytic dataset and shared files. This limited processing was compatible with the approved protocol and informed-consent materials, which permitted research use of anonymized course data. No AI-assigned code entered the reported dataset or results. The pass informed codebook v2. Two human coders subsequently reviewed all 120 records in separate files using v2 and made their coding decisions independently (Campbell et al., 2013; O’Connor & Joffe, 2020). Both coders had the definitions, inclusion and exclusion rules, and example anchors. Comparison of their completed files informed v2.1, which tightened the categories that generated most disagreement: expression safety required evidence about voice or speaking space; low-pressure trial required explicit rehearsal or low-risk use; peer support required an actual contribution; and complementarity required an explicit relation between sources.

Exact agreement was 119/120 (99.2%) for source, 112/120 (93.3%) for function, 96/120 (80.0%) for experience, and 118/120 (98.3%) for boundary codes. Mean Jaccard similarity for the multi-label sets was 0.994, 0.969, 0.854, and 0.988, respectively. All four families matched exactly in 88 cases (73.3%). These values describe agreement on a fixed, multi-label coded corpus; they are not chance-corrected coefficients. The lower agreement for experience codes led to stricter inclusion rules before the codebook was frozen.

For the 32 records with a family-level difference, the conservative consensus retained only codes assigned by both coders; a disputed family with no shared code was recorded as insufficient. One-coder codes remained in the audit file but were excluded from the reported results. This rule favors specificity and may undercount implicit experience. Codebook v2.1 formalized these tightened boundaries and the consensus rule before the final tables were produced. The 120 records were not independently coded a third time after v2.1 was frozen; the same corpus supported both refinement and the final descriptive tables. Agreement values are therefore process diagnostics rather than independent validation of the codebook.

### 3.6. Analytic Strategy and Mixed-Methods Integration

Analysis proceeded in four stages: profiling coverage across seven reflections; using the structured source item as an implementation check within each Reflection 4 and 6 condition; calculating sampling-weighted function and experience percentages within consensus source categories; and interpreting maximum-variation cases. Data management, fixed-seed sampling, agreement summaries, and descriptive tables were produced in Python 3.14.0 with pandas 2.3.3. Sensitivity analyses compared weighted and unweighted estimates and then repeated the AI- and teacher-salient summaries separately at Reflections 4 and 6. Source percentages describe remembered salience, not interaction frequency or exposure. Multi-label percentages do not sum to 100%. Remembered source was not independently assigned, repeated submissions by the same student were not modeled, and the peer and mixed groups were sparse. The weighted analyses were therefore treated as descriptive estimates of the 291-record substantive corpus rather than as inferential comparisons.

Six maximum-variation cases made source-role distinctions inspectable (Palinkas et al., 2015). Selection required exact coder agreement and covered AI initial elaboration, teacher challenge, peer reasoning, an expression constraint, reason organization with an AI-risk boundary, and AI-peer complementarity. The cases illustrate patterns; they do not establish prevalence or condition superiority.

The strands were integrated during interpretation rather than through a combined statistical model. The structured item described the remembered-source profiles observed within each classroom arrangement. Qualitative coding examined the cognitive work and experience attached to those sources, and the case display made the resulting interpretation inspectable. Frequent AI source salience together with reason-organization accounts supported an interpretation of student-reported cognitive engagement. The near absence of low-pressure-trial codes, by contrast, did not support an affective- or participation-engagement claim.

### 3.7. Anonymization, Translation, and Ethics

Quotation candidates underwent privacy and translation review. Identifiers and unnecessary sensitive details were removed. Yuqiang Liu translated the six selected Chinese excerpts into English while preserving first-person stance, degree, uncertainty, and reasoning relations. Zehua Ji independently back-translated the English excerpts and checked them against the Chinese source (Squires, 2009). Raw reflections and case mappings will not be made public because of re-identification risk.

The protocol was prospectively reviewed and approved on 2 March 2026 by the School of Economics and Management, North University of China, acting as the school-level institutional ethics review body, before the first reflection data were collected on 4 March 2026. The formal approving body named on the official institutional record is the School itself; the record names no separate committee and assigns no approval number. Students received information about the study purpose, data use, confidentiality, voluntary participation, and withdrawal. Only anonymized course data from students providing paper or electronic informed consent were analyzed. Non-participation did not affect learning, assessment, course rights, or grades.

## 4. Results

### 4.1. Coverage and Source-Salience Implementation Check

The corpus provided broad course coverage but not a balanced longitudinal record. Of 1034 matched submissions, 648 contained substantive text. Reflections 2, 3, 5, and 7 had uneven condition coverage, so Reflections 4 and 6 provided the common comparison: 399 matched submissions and 291 substantive texts (Table 1). Substantive-response rates in the teacher-led condition were 58.3% at Reflection 4 and 55.9% at Reflection 6, compared with 78.8–79.1% at Reflection 4 and 79.1–89.8% at Reflection 6 in the AI-supported and hybrid conditions. Restricting teacher-led responses to substantive texts reduced the teacher-source share from 76.4% to 73.8% at Reflection 4 and from 61.8% to 55.3% at Reflection 6 (Supplementary Table S6). The intermittent coverage precludes claims about change over time, while the source-profile shift indicates possible underrepresentation of students who selected teacher questioning but supplied minimal open text, especially at Reflection 6.

The structured item served as an implementation check and aligned closely with classroom arrangement (Table 2). At Reflection 4, AI was salient for 67.2% of A and 60.6% of H responses, whereas the teacher was salient for 76.4% of T responses. At Reflection 6, AI accounted for 68.7% in A and 66.1% in H, and the teacher for 61.8% in T. Peer questioning was more visible in T at Reflection 6 (25.0%) than in A (6.0%) or H (6.8%). These profiles show that students remembered distinguishable questioning environments; they are not substantive tests of cognitive engagement or question quality.

In the 120-record coded sample, the final source categories were AI (*n* = 62), teacher (*n* = 45), peer (*n* = 7), mixed (*n* = 4), and classroom/task or other (*n* = 2). After weighting for the six sampling fractions, these categories represented an estimated 55.0%, 35.0%, 5.0%, 3.6%, and 1.4% of the 291-record substantive corpus. The distribution sets the interpretive boundary: AI and teacher patterns can be compared descriptively, whereas peer and mixed patterns are better examined through cases than through percentage differences.

### 4.2. Shared Cognitive Work, Different Points of Emphasis

AI- and teacher-salient reflections overlapped in the cognitive work described (Table 3). In the sampling-weighted estimates, AI-salient reflections most often included value conflict (77.3%), reason asking (58.0%), consequence reasoning (54.3%), assumption testing (50.8%), and clarification (38.6%). Teacher-salient reflections concentrated on value conflict (74.1%), reasons (59.0%), consequences (57.4%), and clarification (50.2%). Both sources were therefore associated with reasons, consequences, and competing values.

The emphases differed. Weighted assumption-testing estimates were 50.8% for AI-salient reflections and 20.6% for teacher-salient reflections; evidence testing was 16.6% and 2.6%; and uncertainty was 18.2% and 4.7%. Teacher-linked reflections showed more clarification. Weighting changed the AI and teacher estimates by no more than 1.9 percentage points and did not reverse these contrasts. Separate Reflection 4 and 6 summaries also retained the AI-greater direction for assumption and evidence codes and the teacher-greater direction for clarification. These are associations within the structured intervention: the AI prompt explicitly requested assumptions and evidence, while teacher questions were generated responsively rather than from the same checklist.

The cases made this distinction visible. In Q1, an AI-salient question replaced an unconditional judgment with attention to costs and conditions. In Q2, a teacher-salient question helped distinguish reasoned disagreement from malicious attack. Q3 linked peer discussion to comparing risk, cost, and fairness. Each case shows a different route into qualification rather than movement toward a predetermined answer.

The seven peer-salient cases were too few for broad comparison. In raw counts, four involved counter-positioning and five involved consequence reasoning; the corresponding sampling-weighted estimates were 55.4% and 67.1%. All seven cases involved value conflict. This concentration is analytically useful but is not prevalence evidence.

### 4.3. Cognitive Organization Was Clearer than Affective Safety

Experience codes qualified the engagement interpretation (Table 4). In the weighted estimates, reason organization appeared in 57.4% of AI-salient reflections, perspective taking in 26.1%, expression safety in 14.5%, and low-pressure trial in 1.7%. Teacher-salient reflections showed the same ordering: reason organization 34.3%, perspective taking 25.8%, and expression safety 10.1%. Weighting changed AI and teacher experience estimates by no more than 1.4 percentage points. The evidence therefore supports cognitive organization more clearly than reduced social or emotional risk.

Q5 described converting an intuitive judgment into a threshold and decision rule, but did not report greater confidence or participation. Q4 concerned constraints on reasonable criticism; it did not show that the student personally spoke more, felt safer, or became less silent. These distinctions prevent cognitive organization and topic-level discussion of expression from being recast as behavioral or emotional engagement gains.

No AI-salient case survived consensus as mechanical or superficial, and no teacher-salient case survived as teacher pressure. These zeros do not establish absence. They mean only that the themes were not retained by both coders under the conservative consensus rule.

### 4.4. Peer Reasoning and Perceived Complementarity

Peer questioning was uncommon as the most salient source, but four of seven peer-salient reflections involved perspective taking and one described collaborative support. These cases position peer talk as a setting for comparing criteria and explaining a position, not as evidence of a general low-pressure mechanism.

The four mixed-source cases were code-rich: all involved perspective taking, three reason organization and low-pressure trial, two peer support, and one AI dependence. Q6 described AI questions and group discussion as complementary resources. Because the reflection did not record a complete sequence, it supports perceived complementarity rather than a verified orchestration mechanism.

The single dependence case made the boundary explicit. The same mixed-source reflection represented by Q5 described clearer criteria but also stated that the group “relied a little too much on AI”. This does not establish a general dependence problem; it shows that reason organization and perceived over-delegation could coexist in one student’s account (Table 5).

### 4.5. Integrated Interpretation: A Differentiated Questioning Ecology

Read together, the structured item, weighted coded frequencies, and cases separate source visibility from the work students associated with it. Students in the three classroom arrangements remembered different question-source profiles, but source salience alone did not demonstrate cognitive engagement. Within this intervention, AI-salient reflections connected that source most clearly to assumptions, missing criteria, evidence, uncertainty, and reason organization. The AI prompt had explicitly solicited several of these functions, so the pattern supports the operation of this questioning design rather than an inherent property of GenAI. Teacher-salient clarification and the smaller set of peer comparisons identified other resources. The sparse mixed-source evidence suggested possible complementarity but did not verify a complete sequence of uptake.

Taken together, the observed associations were differentiated rather than ranked. The evidence does not show H to be more effective than A or T, and it does not support a general affective-safety claim. It supports a narrower account of the reported experience: students described cognitive engagement when responding to structured GenAI questions, while contextual clarification, comparison with others, and final judgment remained distributed across the classroom setting.

## 5. Discussion

### 5.1. Engagement Depends on Cognitive Work, Not Tool Presence

This study began from a simple distinction: access to questions is not the same as engagement with them. Students connected AI, teacher, and peer questions to overlapping cognitive work but different emphases. AI-salient accounts more often concerned assumptions, criteria, evidence, uncertainty, and organizing an initial position. Teacher-salient accounts made situated clarification more visible. The smaller peer set centered on comparison, counter-positioning, consequences, and competing values. Sampling weights did not materially alter these patterns. Similar question functions therefore acquired different meanings through the designed prompt, remembered source, and classroom context.

This finding sharpens how engagement should be discussed in GenAI-supported learning. Engagement is not adequately represented by tool use, favorable attitudes, or participation volume (Fredricks et al., 2004; Kahu, 2013). The reflections showed students generating conditions, qualifications, comparisons, and decision rules, which are consistent with constructive cognitive activity (Chi & Wylie, 2014). They did not show that students became more behaviorally active, emotionally secure, or agentically influential. This boundary matters because recent GenAI research often moves quickly from use or perceived usefulness to claims about engagement. The present evidence supports the quality of remembered reasoning work, not engagement as an undifferentiated outcome.

The source differences also extend dialogic research showing that cognitive demand is distinct from dialogic orientation (Uştuk et al., 2025) and that participation volume does not capture connected reasoning (Bouton et al., 2025). The design question is therefore not which source asks the best question in general, but what cognitive work remains to be done and which source is positioned to support it.

### 5.2. AI Questions Support Elaboration but Do Not Complete the Dialogue

Within this intervention, AI was most visible as a resource for elaborating an initial view. Students described prompts that exposed conditions, requested evidence, or turned an intuition into a more organized reason. This result is partly built into the instructional design: the common AI prompt explicitly requested questions about assumptions, evidence, consequences, and consistency. The analysis therefore shows what became salient when general-purpose systems were assigned that role, not what GenAI necessarily contributes under other prompts. Students still had to decide what to inspect, accept, or reject (Hwang & Lee, 2025; Kim et al., 2025). This pattern is consistent with recent work showing that the educational value of GenAI depends on how it is embedded and whether it supports rather than replaces learner agency (G. Wang et al., 2026). It also supports the broader argument that increasingly capable technology makes the surrounding relational, metacognitive, and ethical work more consequential, not less (Deneen et al., 2026).

AI did not produce dialogue on its own. A plausible prompt may be ignored, answered superficially, or accepted without checking; fluent assistance can weaken monitoring when reasoning is delegated (Fan et al., 2025; Stadler et al., 2024). The single dependence concern marked this boundary without establishing a general problem. Likewise, one low-pressure AI case cannot support claims that AI reduced anxiety or classroom silence. The evidence is stronger for reason elaboration than for expressive safety.

Teacher- and peer-salient accounts supplied work that prompt generation did not. Teacher questions clarified positions in relation to the immediate issue. Peer questions required students to compare costs, risks, and values with another person. These patterns resemble teacher mediation that turns AI output into an object for evaluation (Borges et al., 2026) and research showing that comparison and integration require structured peer interaction (He et al., 2025; J. Wang et al., 2025). The reflections did not capture complete uptake chains, so they cannot show how often teachers connected answers or how often peers changed a subsequent contribution. They show why those human roles remain pedagogically relevant, not that their effects were fully observed.

### 5.3. From Source Substitution to Functional Differentiation

The practical implication is functional rather than technological. A structured AI prompt can follow an initial position by probing assumptions, criteria, evidence, alternatives, or consequences. Peer discussion can compare positions and require explanation. Teacher questioning can clarify what remains unresolved and reconnect the discussion to course purposes. This is not a fixed AI-peer-teacher sequence. The reflections did not show a consistently stronger hybrid pattern, and the four mixed-source cases are too few to establish a model. They instead identify a testable design proposition: complementarity may depend on whether one source’s contribution becomes material for the next stage of reasoning.

This proposition shifts attention from source substitution to instructional connection. One way to test it would be to require students to record which AI question changed their reasoning, justify whether the prompt deserved attention, compare their response with a peer position, and bring unresolved issues into teacher-led discussion. Such routines would keep judgment as student work while making cognitive engagement more inspectable than simply requiring students to open an AI application or generate more prompts.

The case also provides a bounded regional contribution. It shows how widely available Chinese-language AI systems entered one locally organized university course. It does not define a Chinese or Asia-Pacific classroom type. Classroom authority, participation, language, assessment, and access vary across and within countries. Comparative work should test which role distributions travel and which depend on local conditions.

### 5.4. Limitations and Future Research

Several limits follow directly from the design. Reflections were intermittent, and only two points supported a three-condition comparison. The source item measured remembered salience, not question frequency, sequence, or quality. Sampling weights addressed unequal probabilities across the six substantive-text strata, but they could not correct differential entry into that corpus; teacher-led classes had lower substantive-response rates at both common points. Remembered source also remained closely associated with classroom arrangement, and arrangement was partly confounded with programme. The small peer and mixed groups restrict comparison. The reflection prompts themselves invited reason organization, qualification, and perspective taking, so coded content should not be interpreted as spontaneous behavior. The same 120 records informed codebook refinement and final descriptive coding, and conservative consensus may have excluded implicit experiences. The same students could submit more than one reflection, but the analysis did not model change within students. Without complete AI logs, classroom recordings, teacher-discourse coding, or independent fidelity measures, the AI prompt’s contribution cannot be separated from source, and full uptake chains and ignored prompts cannot be reconstructed.

Sex and gender were not analyzed because the approved course-data design did not collect demographic information suitable for defensible subgroup analysis, and the research questions did not posit a sex- or gender-based mechanism. A post hoc subgroup analysis would therefore have exceeded the available data and the study’s analytic purpose.

Future studies should combine reflections with interaction records and compare question sequences while holding topic and function constant. A stronger test would examine whether an AI prompt leads to a student explanation, whether a peer or teacher takes up that explanation, and whether the student later revises or transfers the reasoning. Outcomes should distinguish behavioral entry into discussion, cognitive quality, emotional experience, agentic contribution, uptake by others, and later performance rather than merging them under a single engagement label.

## 6. Conclusions

Within this structured intervention, students reported AI most clearly in connection with elaborating positions, teachers with situated clarification, and peers with a small set of comparative moves. The clearest reported engagement signal was cognitive organization, not greater participation or emotional safety. These accounts do not establish inherent source effects, critical-thinking gains, complete uptake sequences, or hybrid-condition superiority. They support a testable design proposition for future study: assigning sources distinct reasoning tasks may be useful when students must evaluate how one contribution informs the next. The educational question is not whether GenAI can generate a plausible prompt, but whether students use that prompt to examine and revise their own judgment.

## Figures and Tables

**Table 1:** Reflection-card coverage and analytic role.

Reflection	Reflection-Card Focus	Raw	Matched	Unique Students	Substantive	Matched A/H/T	Substantive A/H/T	Analytic Role
1	Course orientation and initial expectations	177	172	171	69	69/67/36	30/27/12	Background only; source item not yet salient.
2	Short-video credibility	75	73	72	44	1/0/72	1/0/43	Additional T-condition media-credibility exercise; not the common Week 2 task and not used for three-condition comparison.
3	Trolley problem and autonomous-vehicle ethics	152	140	138	104	71/69/0	46/58/0	A/H comparison; no T coverage.
4	Campus speech boundaries and free expression	211	205	205	147	67/66/72	53/52/42	Core common comparison point.
5	Online anonymity and responsibility	65	64	64	41	64/0/0	41/0/0	Local A-condition case; not used for three-condition comparison.
6	Public-health measures and individual choice	199	194	193	144	67/59/68	53/53/38	Core common comparison point.
7	Environmental policy and intergenerational fairness	193	186	185	99	68/49/69	36/26/37	Supplementary late-course evidence; H coverage lower.
Total		1072	1034	221	648	407/310/317	260/216/172	

Note. A/H/T values are reported in that order. The total of 648 refers to matched eligible first-year records; 670 of the 1072 raw submissions met the substantive-text rule before roster matching. Reflection numbers identify collection instruments, not a one-to-one weekly sequence. Reflection 2 was an additional T-condition short-video credibility exercise and was not used for three-condition comparison.

**Table 2:** Remembered question sources by reflection point and condition.

Reflection	Condition	n Matched	AI	Teacher	Peer	Other
4	A	67	45 (67.2%)	21 (31.3%)	1 (1.5%)	0 (0.0%)
4	H	66	40 (60.6%)	23 (34.8%)	3 (4.5%)	0 (0.0%)
4	T	72	7 (9.7%)	55 (76.4%)	7 (9.7%)	3 (4.2%)
6	A	67	46 (68.7%)	17 (25.4%)	4 (6.0%)	0 (0.0%)
6	H	59	39 (66.1%)	16 (27.1%)	4 (6.8%)	0 (0.0%)
6	T	68	8 (11.8%)	42 (61.8%)	17 (25.0%)	1 (1.5%)

Note. Percentages are within each reflection-condition cell and use all matched responses, including responses without substantive open text. They describe students’ remembered salient source, not observed exposure, question frequency, or question quality.

**Table 3:** Questioning functions by remembered source.

Source	Raw *n*	Weighted *N*	Clarification	Reasons	Evidence	Assumptions	Counter-Positioning	Consequences	Value Conflict	Uncertainty	Expression
AI	62	160.0	24 (38.6%)	36 (58.0%)	10 (16.6%)	31 (50.8%)	20 (31.8%)	34 (54.3%)	48 (77.3%)	11 (18.2%)	14 (22.9%)
Teacher	45	101.8	23 (50.2%)	26 (59.0%)	1 (2.6%)	9 (20.6%)	11 (26.3%)	26 (57.4%)	33 (74.1%)	2 (4.7%)	7 (15.2%)
Peer	7	14.4	1 (13.1%)	1 (13.1%)	0 (0.0%)	1 (13.1%)	4 (55.4%)	5 (67.1%)	7 (100.0%)	0 (0.0%)	1 (14.5%)
Mixed	4	10.6	4 (100.0%)	3 (74.9%)	1 (25.1%)	3 (74.9%)	3 (74.9%)	4 (100.0%)	3 (74.9%)	0 (0.0%)	1 (24.6%)
Classroom/task or other	2	4.2	1 (50.0%)	1 (50.0%)	0 (0.0%)	0 (0.0%)	0 (0.0%)	1 (50.0%)	1 (50.0%)	0 (0.0%)	1 (50.0%)

Note. Cells report unweighted code counts followed by sampling-weighted percentages. Weighted *N* is the sum of *N_h/n_h* weights within each source category and estimates the 291-record substantive corpus. Coding was multi-label, so percentages do not sum to 100%. Peer, mixed, and other groups remain too small for stable percentage comparison. Unweighted percentages and reflection-stratified sensitivity estimates appear in Supplementary Tables S7 and S9.

**Table 4:** Student-reported experience themes by remembered source.

**Panel A.** Reported engagement experiences.							
**Source**	**Raw *n***	**Weighted *N***	**Expression** **Safety**	**Low-Pressure** **Trial**	**Perspective** **Taking**	**Reason** **Organization**	**Continued** **Questioning**
AI	62	160.0	9 (14.5%)	1 (1.7%)	16 (26.1%)	35 (57.4%)	0 (0.0%)
Teacher	45	101.8	5 (10.1%)	0 (0.0%)	11 (25.8%)	15 (34.3%)	2 (4.6%)
Peer	7	14.4	0 (0.0%)	0 (0.0%)	4 (55.4%)	1 (13.1%)	0 (0.0%)
Mixed	4	10.6	1 (24.6%)	3 (74.9%)	4 (100.0%)	3 (74.9%)	0 (0.0%)
Classroom/task or other	2	4.2	0 (0.0%)	0 (0.0%)	0 (0.0%)	1 (50.0%)	0 (0.0%)
**Panel B.** Reported limits and role boundaries.							
**Source**	**Mechanical**		**Superficial**		**AI Dependence**	**Teacher Pressure**	**Peer Support**
AI	0 (0.0%)		0 (0.0%)		0 (0.0%)	0 (0.0%)	0 (0.0%)
Teacher	0 (0.0%)		0 (0.0%)		0 (0.0%)	0 (0.0%)	0 (0.0%)
Peer	0 (0.0%)		0 (0.0%)		0 (0.0%)	0 (0.0%)	1 (13.1%)
Mixed	0 (0.0%)		0 (0.0%)		1 (25.1%)	0 (0.0%)	2 (49.8%)
Classroom/task or other	0 (0.0%)		0 (0.0%)		0 (0.0%)	0 (0.0%)	0 (0.0%)

Note. Cells report unweighted code counts followed by sampling-weighted percentages. Coding was multi-label. Zero means that no case in the coded sample survived the two-coder conservative consensus rule for that code; it does not establish absence from the full corpus. Peer, mixed, and other groups remain too small for stable percentage comparison. Unweighted percentages and reflection-stratified sensitivity estimates appear in Supplementary Tables S8 and S9.

**Table 5:** Audited maximum-variation cases.

Quote	Salient Source Role	Analytic Pattern	Audited English Excerpt	Interpretive Boundary
Q1	AI	Initial elaboration of consequences and trade-offs	“I used to think that stricter controls were necessarily safer, but this discussion made me reconsider… I came to see that there is no perfect choice: every option carries costs, and the issue cannot be settled by a simple one-size-fits-all judgement.”	Illustrates conditional reasoning; does not show that AI was superior to teacher questioning.
Q2	Teacher	Situated clarification and second-stage challenge	“I had initially thought that different voices should be respected without condition, but this question made me realise that reasoned discussion must be distinguished from malicious attack.”	Illustrates position refinement in one teacher-salient case.
Q3	Peer	Collaborative counter-positioning and reason organisation	“Today’s small-group discussion moved me beyond an either-or judgement and taught me to consider the issue through several dimensions: risk, cost, and fairness.”	Peer-source cases were few; the excerpt is explanatory rather than representative.
Q4	Mixed	Perceived expressive consequence	“Overly strict deletion standards would narrow the space for students to make reasonable criticisms of public affairs and make people reluctant to speak.”	Concerns perceived constraints on voice; it does not measure actual participation or personal psychological safety.
Q5	Mixed	Operational criteria and an AI-dependence boundary	“When I was asked, ‘How high counts as high, and who gets to decide?’, I realised that I had no workable threshold at all. A position without boundary conditions is only a statement, not a principle.”	The same reflection also raised a mild dependence concern; neither theme supports a general prevalence claim.
Q6	Mixed	AI-peer complementarity	“Today’s AI follow-up questions and small-group discussion moved me beyond black-and-white thinking and helped me consider public policy from the positions of different groups and across different dimensions of risk.”	Shows perceived complementarity, not the superiority of the hybrid condition.

Note. Quote labels are publication-facing analytic labels. Internal case identifiers, class codes, subgroup labels, and exact submission times were removed. Quotations were translated from Chinese and independently checked by back-translation for stance, degree, and reasoning relations.

## Data Availability

The anonymized 120-case consensus coding matrix and aggregate source-salience implementation-check data are provided as Supplementary Data S1. The frozen codebook and analytic rules are included in the Supplementary Material. Additional de-identified materials may be requested from the corresponding author, subject to institutional review of the proposed use and applicable privacy safeguards. Raw reflection text, student identifiers, class and subgroup mappings, coder memos, and quotation-to-case links are not shared because combinations of these fields could permit re-identification.

## Supplementary Materials

The following supporting information can be downloaded at: https://www.mdpi.com/article/10.3390/bs16101814/s1, Supplementary Table S1: Full reflection codebook; Supplementary Methods S1: Coding and conservative consensus; Supplementary Table S2: Tightened inclusion and exclusion rules; Supplementary Methods S2: Anonymization and translation; Supplementary Methods S3: Standard AI question-partner prompt; Supplementary Table S3: Weeks 2–7 issue-based tasks; Supplementary Table S4: Reflection-card field and construct map; Supplementary Table S5: Anonymized class and instructor allocation; Supplementary Table S6: Coverage, selection, and sampling weights; Supplementary Table S7: Unweighted and sampling-weighted questioning-function estimates; Supplementary Table S8: Unweighted and sampling-weighted experience estimates; Supplementary Table S9: Reflection-stratified AI- and teacher-salient sensitivity estimates; Supplementary Data S1: Anonymized coding matrix and table provenance. Supplementary Data S1 is supplied as a separate machine-readable ZIP archive. No raw reflection text, student identifiers, class or subgroup mappings, coder memos, or quotation-to-case links are included in the shared data.
