# Supplementary material for: Student-Reported Cognitive Engagement with Structured Generative-AI Questioning: Differentiated Associations with AI, Teacher, and Peer Questions
Source: Behav Sci (Basel). 2026 Oct 4;16(10):1814. doi: 10.3390/bs16101814 (PMC13701548; doi:10.3390/bs16101814)
Supplement: Supplementary file 1 [file behavsci-16-01814-s001.zip › behavsci-4567673-supplementary.pdf]

Supplementary material

Student-Reported Cognitive Engagement with Structured Generative-AI Questioning: Differentiated Associations with AI, Teacher, and Peer Questions

Supplementary Table S1. Full reflection codebook

Family	Code	Operational definition
Source	AI	The salient question is attributed to an AI system.
Source	Teacher	The salient question is attributed to a teacher.
Source	Peer	A classmate or small group performs an identifiable questioning, explanatory, or complementary role.
Source	Classroom/task	The topic or task is salient without a specific human or AI source.
Source	Mixed/unclear	Two or more sources are comparably salient and no single source dominates.
Function	Clarification	Defines a term, standard, threshold, or boundary.
Function	Reason asking	Requests or develops reasons and justification.
Function	Evidence testing	Requests evidence, facts, or a test of a claim.
Function	Assumption testing	Exposes or examines an unstated premise.
Function	Counter-positioning	Introduces an opposing position, alternative, or difficult case.
Function	Consequence reasoning	Traces likely effects, costs, or risks.
Function	Value conflict	Makes an explicit tension between values, interests, or burdens visible.
Function	Uncertainty	Marks doubt, conditionality, or limits to a judgment.
Function	Expression	Prompts speaking, voice, or reflection on the space to express a view.
Experience	Expression safety	Explicitly concerns willingness or space to speak; policy-level safety alone is excluded.
Experience	Low-pressure trial	AI or informal peer discussion is explicitly used to try out or rehearse a response.
Experience	Multi-perspective	The student reports seeing another position or angle.
Experience	Reason organization	Questioning helps organize reasons, criteria, or a principle.
Experience	Continue questioning	The student explicitly wants further questioning or discussion.
Experience	Mechanical	AI questioning is experienced as templated or decontextualised.
Experience	Superficial	Questioning is experienced as insufficiently deep.
Experience	Dependence	The student expresses concern about reliance on AI.
Experience	Teacher pressure	The student reports pressure or anxiety linked to teacher questioning.
Experience	Peer collaborative support	Peer discussion provides explicit explanatory, complementary, or social support.
Boundary	AI initial	AI is positioned as an initial follow-up or elaboration resource.
Boundary	Teacher second-stage	Teacher judgment is positioned as a deeper or situated follow-up resource.
Boundary	Peer social	Peer questioning is positioned as collaborative explanation or social reasoning.
Boundary	AI risk	The text identifies mechanical, superficial, dependence, or context-loss risk.
Boundary	Orchestration	At least two sources are linked through sequencing, complementarity, or role division.

Note. Coding was multi-label. Function and experience codes required explicit textual evidence; topic content alone was insufficient.

Supplementary Methods S1. Coding and conservative consensus

Codebook v1 was developed from the research questions, reflection fields, and an initial reading of the corpus. An AI-assisted preparatory pass used de-identified Chinese excerpts to flag ambiguous boundaries and organize a disagreement template; no AI-assigned code entered the reported data or results. This pass informed codebook v2. Two human coders then reviewed all 120 Chinese-language records in separate files using v2 and made their decisions independently. Exact agreement was 99.2% for source, 93.3% for function, 80.0% for experience, and 98.3% for boundary codes; mean Jaccard similarity was 0.994, 0.969, 0.854, and 0.988, respectively. Comparison of the completed files informed v2.1. For 32 records with at least one family-level difference, the conservative consensus retained only codes shared by both coders; a family with no shared code was recorded as insufficient. The 120 records were not independently coded a third time after v2.1 was frozen. The agreement values are therefore process diagnostics rather than independent codebook validation.

Supplementary Table S2. Tightened inclusion and exclusion rules

Code	Include when the text shows	Exclude when the text shows only
Peer source	A peer or group asks, explains, adds, compares, or prompts another view.	Peers are only people affected by the issue or receive a general classroom evaluation.
Value conflict	Two values, interests, rights, burdens, or costs are placed in tension.	The topic is value-laden without both sides being named.
Expression safety	Willingness, room, or constraint to speak or give a genuine view.	Safety refers to health, risk, control, or policy.
Low-pressure trial	An explicit space to try, rehearse, or organize an answer with low risk.	AI is merely present or the student only reports rethinking.
Peer support	Peer discussion provides explanatory, complementary, or social support.	A peer source is named without a support experience.
AI dependence	The student expresses reliance on AI.	Dependence concerns a non-AI object.
Orchestration	At least two sources are linked through sequence, complementarity, coordination, or role division.	Several sources are present without a relation between their roles.

Supplementary Methods S2. Anonymization and translation

Names, student numbers, original class codes, subgroup labels, and exact submission times were removed before coding. Published excerpts use Q1-Q6. Sensitive third-party details were omitted when unnecessary. Yuqiang Liu translated the six selected Chinese excerpts into English while preserving first-person stance, degree, uncertainty, and reasoning relations. Zehua Ji independently back-translated the English excerpts and checked them against the Chinese source. Raw reflections and quotation-to-case mappings are not public because of re-identification risk.

Supplementary Methods S3. Standard AI question-partner prompt

The weekly topic changed, while the role and follow-up constraints remained stable. The common prompt is reproduced below.

Chinese original

你现在扮演一位苏格拉底式的思辨导师。我们是一组大学新生，正在讨论一个问题。下面我会告诉你我们目前的主张和理由。你的任务是：1. 只用问题来回应我们，不要直接给出你的结论或建议；2. 尤其要追问我们的假设、证据、可能忽略的后果，以及不同情境下我们立场是否自洽；3. 每次最多提出 2-3 个问题，然后等待我们回答，再根据我们的回答继续提问；4. 如果我们给出的回答太含糊或太空泛，请继续追问，让我们说得更具体一些。我们当前的主张和理由是（由小组填写后输入）：[在此输入本组立场和 1-2 条理由]。

English translation

You are acting as a Socratic thinking mentor. We are a group of first-year undergraduates discussing an issue. We will give you our current claim and reasons. Your task is to: (1) respond only with questions and not provide your own conclusion or advice; (2) ask follow-up questions about our assumptions, evidence, possible overlooked consequences, and whether our position remains consistent across different situations; (3) ask no more than two or three questions at a time, wait for our answer, and then continue questioning in response; and (4) continue asking for detail if our answer is vague or too general. The group then enters its position and one or two reasons.

Supplementary Table S3. Weeks 2-7 issue-based tasks

Week	Classroom issue	Main reasoning task
2	AI writing tools in university assignments	Take a position, give reasons, consider an opposing view, and identify uncertainty.
3	Trolley, tunnel, and autonomous-vehicle ethics	Compare choices, state a principle, test consistency, and consider objections.
4	Campus speech boundaries and responsibility	Set a boundary standard, compare roles, anticipate effects, and answer objections.
5	Online anonymity and responsibility	Balance speaker protection and harm prevention across boundary cases.
6	Public-health measures and individual freedom	Justify proportional restrictions and compare burdens across groups.
7	Environmental policy and intergenerational fairness	Propose a policy package and compare present costs with future benefits.

Note. The table summarizes the common Weeks 2-7 intervention tasks preserved in the archived teaching plans. Reflection numbers identify collection instruments and do not map one-to-one onto this sequence. Reflection 2 in main-text Table 1 was an additional teacher-condition card on short-video credibility rather than the common Week 2 AI-writing task. The table contains no student responses.

Supplementary Table S4. Reflection-card field and construct map

Recurring field	English rendering of the recurring prompt	Analytic use	Evidence boundary
Question source	Which source asked the question that made you think most: AI, teacher, peer, or other?	Remembered source salience	Self-report; not observed exposure, frequency, or quality
Question recall	Write the question as closely as possible to its original wording.	Textual anchor for source and function coding	Recall may be selective or paraphrased
Why it mattered	Why did this question make you think, and what did it prompt you to examine?	Question function and reported cognitive work	Retrospective account; not a direct process trace
Position and change	State your current position and explain what changed or became more conditional, if anything.	Qualification, uncertainty, and reason organization	Does not establish durable learning or critical-thinking gain
Alternative standpoint	Identify another standpoint, group, or situation that should be considered.	Counter-positioning and perspective taking	Prompt wording varied with the weekly issue
Activity comment	What else would you like to say about the topic, questioning process, or peer discussion?	Experience, risk, and perceived complementarity	Optional comments were uneven in length and detail

Note. The first reflection followed course orientation and was used for background only. Later cards retained these recurring functions while adapting examples and one or two issue-specific prompts to the weekly topic.

Supplementary Table S5. Anonymized class and instructor allocation

Programme	Anonymous class	Condition	Matched reflection contributors	Instructor
Programme 1	P1-C1	A	39	Yuqiang Liu
Programme 1	P1-C2	A	38	Yuqiang Liu
Programme 1	P1-C3	T	36	Yuqiang Liu
Programme 2	P2-C1	T	37	Yuqiang Liu
Programme 2	P2-C2	H	35	Yuqiang Liu
Programme 2	P2-C3	H	36	Yuqiang Liu

Note. Contributor counts refer to first-year students matched to at least one reflection submission. Original class identifiers are withheld. A, H, and T denote AI-supported, hybrid, and teacher-led questioning arrangements. The same instructor taught all six classes using the common course materials.

Supplementary Table S6. Coverage, selection, and sampling weights

Reflection	Condition	Matched n	Substantive n (%)	Sampling weight	All matched source profile	Substantive source profile
4	A	67	53 (79.1%)	2.65	67.2/31.3/1.5/0.0	77.4/20.8/1.9/0.0
4	H	66	52 (78.8%)	2.6	60.6/34.8/4.5/0.0	65.4/32.7/1.9/0.0
4	T	72	42 (58.3%)	2.1	9.7/76.4/9.7/4.2	7.1/73.8/14.3/4.8
6	A	67	53 (79.1%)	2.65	68.7/25.4/6.0/0.0	77.4/18.9/3.8/0.0
6	H	59	53 (89.8%)	2.65	66.1/27.1/6.8/0.0	69.8/24.5/5.7/0.0
6	T	68	38 (55.9%)	1.9	11.8/61.8/25.0/1.5	15.8/55.3/28.9/0.0

Note. Source profiles report AI/teacher/peer/other percentages. Each coded record was weighted by N_h/n_h within its Reflection 4/6 by A/H/T stratum. Sampling weights correct unequal sampling fractions after entry into the substantive-text frame; they do not correct differential substantive-response rates.

Supplementary Table S7. Unweighted and sampling-weighted questioning-function estimates

Source	Raw n	Weighted N	Function	Raw count	Unweighted %	Weighted %	Change (pp)
AI	62	160.0	Clarification	24	38.7	38.6	-0.1
AI	62	160.0	Reason asking	36	58.1	58.0	-0.0
AI	62	160.0	Evidence testing	10	16.1	16.6	0.4
AI	62	160.0	Assumption testing	31	50.0	50.8	0.8
AI	62	160.0	Counter-positioning	20	32.3	31.8	-0.4
AI	62	160.0	Consequence reasoning	34	54.8	54.3	-0.5
AI	62	160.0	Value conflict	48	77.4	77.3	-0.1
AI	62	160.0	Uncertainty	11	17.7	18.2	0.5
AI	62	160.0	Expression	14	22.6	22.9	0.3
Teacher	45	101.8	Clarification	23	51.1	50.2	-0.9
Teacher	45	101.8	Reason asking	26	57.8	59.0	1.2
Teacher	45	101.8	Evidence testing	1	2.2	2.6	0.4
Teacher	45	101.8	Assumption testing	9	20.0	20.6	0.6
Teacher	45	101.8	Counter-positioning	11	24.4	26.3	1.9
Teacher	45	101.8	Consequence reasoning	26	57.8	57.4	-0.4
Teacher	45	101.8	Value conflict	33	73.3	74.1	0.7
Teacher	45	101.8	Uncertainty	2	4.4	4.7	0.2
Teacher	45	101.8	Expression	7	15.6	15.2	-0.3
Peer	7	14.4	Clarification	1	14.3	13.1	-1.1
Peer	7	14.4	Reason asking	1	14.3	13.1	-1.1
Peer	7	14.4	Evidence testing	0	0.0	0.0	0.0
Peer	7	14.4	Assumption testing	1	14.3	13.1	-1.1
Peer	7	14.4	Counter-positioning	4	57.1	55.4	-1.8
Peer	7	14.4	Consequence reasoning	5	71.4	67.1	-4.3
Peer	7	14.4	Value conflict	7	100.0	100.0	-0.0
Peer	7	14.4	Uncertainty	0	0.0	0.0	0.0
Peer	7	14.4	Expression	1	14.3	14.5	0.2
Mixed	4	10.6	Clarification	4	100.0	100.0	0.0
Mixed	4	10.6	Reason asking	3	75.0	74.9	-0.1
Mixed	4	10.6	Evidence testing	1	25.0	25.1	0.1
Mixed	4	10.6	Assumption testing	3	75.0	74.9	-0.1
Mixed	4	10.6	Counter-positioning	3	75.0	74.9	-0.1
Mixed	4	10.6	Consequence reasoning	4	100.0	100.0	0.0
Mixed	4	10.6	Value conflict	3	75.0	74.9	-0.1
Mixed	4	10.6	Uncertainty	0	0.0	0.0	0.0
Mixed	4	10.6	Expression	1	25.0	24.6	-0.4
Classroom/other	2	4.2	Clarification	1	50.0	50.0	0.0
Classroom/other	2	4.2	Reason asking	1	50.0	50.0	0.0
Classroom/other	2	4.2	Evidence testing	0	0.0	0.0	0.0
Classroom/other	2	4.2	Assumption testing	0	0.0	0.0	0.0
Classroom/other	2	4.2	Counter-positioning	0	0.0	0.0	0.0
Classroom/other	2	4.2	Consequence reasoning	1	50.0	50.0	0.0

Source	Raw n	Weighted N	Function	Raw count	Unweighted %	Weighted %	Change (pp)
Classroom/other	2	4.2	Value conflict	1	50.0	50.0	0.0
Classroom/other	2	4.2	Uncertainty	0	0.0	0.0	0.0
Classroom/other	2	4.2	Expression	1	50.0	50.0	0.0

Note. Coding was multi-label, so percentages do not sum to 100%. Peer, mixed, and other groups remain too small for stable percentage comparison.

Supplementary Table S8. Unweighted and sampling-weighted experience estimates

Source	Raw n	Weighted N	Experience theme	Raw count	Unweighted %	Weighted %	Change (pp)
AI	62	160.0	Expression safety	9	14.5	14.5	-0.1
AI	62	160.0	Low-pressure trial	1	1.6	1.7	0.0
AI	62	160.0	Perspective taking	16	25.8	26.1	0.3
AI	62	160.0	Reason organization	35	56.5	57.4	0.9
AI	62	160.0	Continued questioning	0	0.0	0.0	0.0
AI	62	160.0	Mechanical	0	0.0	0.0	0.0
AI	62	160.0	Superficial	0	0.0	0.0	0.0
AI	62	160.0	AI dependence	0	0.0	0.0	0.0
AI	62	160.0	Teacher pressure	0	0.0	0.0	0.0
AI	62	160.0	Peer collaborative support	0	0.0	0.0	0.0
Teacher	45	101.8	Expression safety	5	11.1	10.1	-1.0
Teacher	45	101.8	Low-pressure trial	0	0.0	0.0	0.0
Teacher	45	101.8	Perspective taking	11	24.4	25.8	1.4
Teacher	45	101.8	Reason organization	15	33.3	34.3	1.0
Teacher	45	101.8	Continued questioning	2	4.4	4.6	0.2
Teacher	45	101.8	Mechanical	0	0.0	0.0	0.0
Teacher	45	101.8	Superficial	0	0.0	0.0	0.0
Teacher	45	101.8	AI dependence	0	0.0	0.0	0.0
Teacher	45	101.8	Teacher pressure	0	0.0	0.0	0.0
Teacher	45	101.8	Peer collaborative support	0	0.0	0.0	0.0
Peer	7	14.4	Expression safety	0	0.0	0.0	0.0
Peer	7	14.4	Low-pressure trial	0	0.0	0.0	0.0
Peer	7	14.4	Perspective taking	4	57.1	55.4	-1.8
Peer	7	14.4	Reason organization	1	14.3	13.1	-1.1
Peer	7	14.4	Continued questioning	0	0.0	0.0	0.0
Peer	7	14.4	Mechanical	0	0.0	0.0	0.0
Peer	7	14.4	Superficial	0	0.0	0.0	0.0
Peer	7	14.4	AI dependence	0	0.0	0.0	0.0
Peer	7	14.4	Teacher pressure	0	0.0	0.0	0.0
Peer	7	14.4	Peer collaborative support	1	14.3	13.1	-1.1
Mixed	4	10.6	Expression safety	1	25.0	24.6	-0.4
Mixed	4	10.6	Low-pressure trial	3	75.0	74.9	-0.1
Mixed	4	10.6	Perspective taking	4	100.0	100.0	0.0
Mixed	4	10.6	Reason organization	3	75.0	74.9	-0.1
Mixed	4	10.6	Continued questioning	0	0.0	0.0	0.0
Mixed	4	10.6	Mechanical	0	0.0	0.0	0.0
Mixed	4	10.6	Superficial	0	0.0	0.0	0.0
Mixed	4	10.6	AI dependence	1	25.0	25.1	0.1
Mixed	4	10.6	Teacher pressure	0	0.0	0.0	0.0
Mixed	4	10.6	Peer collaborative support	2	50.0	49.8	-0.2
Classroom/other	2	4.2	Expression safety	0	0.0	0.0	0.0
Classroom/other	2	4.2	Low-pressure trial	0	0.0	0.0	0.0

Source	Raw n	Weighted N	Experience theme	Raw count	Unweighted %	Weighted %	Change (pp)
Classroom/other	2	4.2	Perspective taking	0	0.0	0.0	0.0
Classroom/other	2	4.2	Reason organization	1	50.0	50.0	0.0
Classroom/other	2	4.2	Continued questioning	0	0.0	0.0	0.0
Classroom/other	2	4.2	Mechanical	0	0.0	0.0	0.0
Classroom/other	2	4.2	Superficial	0	0.0	0.0	0.0
Classroom/other	2	4.2	AI dependence	0	0.0	0.0	0.0
Classroom/other	2	4.2	Teacher pressure	0	0.0	0.0	0.0
Classroom/other	2	4.2	Peer collaborative support	0	0.0	0.0	0.0

Note. Zero means that no sampled case survived the conservative two-coder consensus rule for that code; it does not establish absence from the full corpus.

Supplementary Table S9. Reflection-stratified AI- and teacher-salient sensitivity estimates

Panel A. Questioning functions

Reflection	Function	AI raw n	Teacher raw n	AI weighted %	Teacher weighted %	AI minus teacher (pp)
4	Clarification	30	24	40.3	59.0	-18.7
4	Reason asking	30	24	69.8	67.7	2.1
4	Evidence testing	30	24	3.4	0.0	3.4
4	Assumption testing	30	24	37.1	8.5	28.6
4	Counter-positioning	30	24	12.8	19.1	-6.2
4	Consequence reasoning	30	24	20.1	34.4	-14.3
4	Value conflict	30	24	79.7	83.8	-4.0
4	Uncertainty	30	24	10.2	3.8	6.3
4	Expression	30	24	43.5	24.7	18.8
6	Clarification	32	21	37.0	39.9	-2.8
6	Reason asking	32	21	46.8	48.8	-2.0
6	Evidence testing	32	21	29.2	5.7	23.5
6	Assumption testing	32	21	63.9	34.9	28.9
6	Counter-positioning	32	21	50.0	34.9	15.1
6	Consequence reasoning	32	21	87.0	84.6	2.5
6	Value conflict	32	21	75.0	62.6	12.4
6	Uncertainty	32	21	25.9	5.7	20.2
6	Expression	32	21	3.2	4.1	-0.8

Panel B. Student-reported experience themes

Reflection	Experience theme	AI raw n	Teacher raw n	AI weighted %	Teacher weighted %	AI minus teacher (pp)
4	Expression safety	30	24	29.6	15.2	14.3
4	Low-pressure trial	30	24	0.0	0.0	0.0
4	Perspective taking	30	24	9.5	22.9	-13.4
4	Reason organization	30	24	59.7	37.2	22.5
4	Continued questioning	30	24	0.0	8.5	-8.5
4	Mechanical	30	24	0.0	0.0	0.0
4	Superficial	30	24	0.0	0.0	0.0
4	AI dependence	30	24	0.0	0.0	0.0
4	Teacher pressure	30	24	0.0	0.0	0.0
4	Peer collaborative support	30	24	0.0	0.0	0.0
6	Expression safety	32	21	0.0	4.1	-4.1
6	Low-pressure trial	32	21	3.2	0.0	3.2
6	Perspective taking	32	21	42.1	29.3	12.9
6	Reason organization	32	21	55.1	30.9	24.2
6	Continued questioning	32	21	0.0	0.0	0.0
6	Mechanical	32	21	0.0	0.0	0.0
6	Superficial	32	21	0.0	0.0	0.0
6	AI dependence	32	21	0.0	0.0	0.0
6	Teacher pressure	32	21	0.0	0.0	0.0

Reflection	Experience theme	AI raw n	Teacher raw n	AI weighted %	Teacher weighted %	AI minus teacher (pp)
6	Peer collaborative support	32	21	0.0	0.0	0.0

Note. These are descriptive sensitivity estimates. Topic-related variation in absolute frequencies remains visible; the analysis does not estimate inherent source or treatment effects.

Supplementary Data S1. Anonymized coding matrix and table provenance

Supplementary Data S1 contains the 120-case anonymized consensus coding matrix with stratum weights, the six aggregate source-salience cells, anonymized class allocation, substantive-response and source-composition checks, weighted function and experience estimates, reflection-stratified sensitivity results, and a provenance readme. The files exclude raw reflections, student identifiers, original class or subgroup mappings, exact times, coder memos, one-coder-only codes, and quotation links.
